# Integrating interventions supported by development assistance for health into local health system: evidence from a China–World Bank–UK rural health system strengthening project (1998–2007)

**DOI:** 10.1136/bmjgh-2023-012853

**Published:** 2024-05-24

**Authors:** Aidan Huang, Yingxi Zhao, Chunkai Cao, Mohan Lyu, Kun Tang

**Affiliations:** 1 Tsinghua University, Beijing, China; 2 Vanke School of Public Health, Tsinghua University, Beijing, China; 3 Nuffield Department of Medicine, Oxford University, Oxford, Oxfordshire, UK; 4 Duke Global Health Institute, Duke University, Durham, North Carolina, USA

**Keywords:** Health policy, Health systems, Public Health, Qualitative study

## Abstract

**Introduction:**

To empirically investigate sustainability of development assistance for health (DAH), we conducted a retrospective case study on the Basic Health Services Project (BHSP) for rural health system strengthening, supported by the World Bank and the UK in China between 1998 and 2007. Specifically, this study examines the integration of the BHSP interventions into China’s health system.

**Methods:**

From December 2021 to December 2022, we reviewed 64 published papers and project or policy documents, and conducted semistructured interviews with 22 key informants, ranging from managers of donor agencies and the government to township-level hospital directors. From February to March 2023, the data were analysed under an analytical framework for integration of targeted health interventions into health systems.

**Results:**

Evidence of the BHSP shows that the integration outcomes can vary by the levels of integration (national or subnational), geographical coverage (project areas or both project and non-project areas) and approach to integration (policy or routinisation). The country’s health system reform facilitated the integration of the interventions relevant to the reform policies, as the BHSP was one of the pilot schemes. However, interventions incompatible with this broad context were integrated to a limited extent. This integration occurred through embedding the project within the existing system, with a higher degree of embeddedness leading to smoother integration. Cross-sectoral leading groups and a technical support system heightened the project visibility and enabled contextualised local adaptation, contributing to the smooth integration of the project interventions.

**Conclusion:**

The DAH-supported interventions can achieve sustainability by being integrated into the local health system. This integration can take various forms to improve health outcomes, including being accepted and internalised, modified as well as innovated and expanded. The host country and development partners can promote DAH sustainability by contextually integrating these interventions within the project scope.

**WHAT IS ALREADY KNOWN ON THIS TOPIC**

- The existing literature on the transition of development assistance for health (DAH) primarily concentrates on changes in the eligibility status of recipients and the continuation of programmes or DAH-supported interventions. However, the concept of DAH sustainability remains elusive and paradoxical.

**WHAT THIS STUDY ADDS**

- This study values DAH-supported interventions that build health systems over time and views DAH sustainability as the improvement of health outcomes.
- Using the case of the China–World Bank–UK Basic Health Services Project in China (1998–2007), the study contributes to the assessment of the integration of DAH-supported interventions into the local health system by independent researchers through the lens of global health.
- The study offers insights on how various contextual factors and intervention characteristics can affect the integration of interventions, and examines ‘one office, two functions’ arrangement and cofinancing as approaches to embedding the project into the existing system. Additionally, it emphasises the importance of increasing project visibility and promoting mutual learning among international and local partners.

**HOW THIS STUDY MIGHT AFFECT RESEARCH, PRACTICE OR POLICY**

- Stakeholders of the host country and development partners can promote DAH sustainability within the project scope by (1) assessing the conducive and obstructive contextual factors and the potential for policy convergence, (2) carefully designing the intervention characteristics while integrating the intervention in the form that is the most beneficial for and compatible with the local health system, (3) determining the level of project embeddedness within the existing system based on the local health system’s characteristics, (4) cultivating project visibility among key decision-makers and (5) promoting dynamic local adaptation through international–local expert collaboration.

## Introduction

Development assistance for health (DAH) encompasses the financial and non-financial resources channelled through international development agencies to support health initiatives in low-income and middle-income countries.^1^ While DAH is intended to reduce global health inequities, it has faced criticism over its long-term sustainability.^2^ The challenge of ensuring DAH sustainability stems from the asymmetric power dynamics between donors and recipients,^3^ which become particularly evident when a country starts to reduce its dependency on donor support^4–6^ and strives towards self-sustaining health programmes.^7 8^ An increasing number of donors adopt DAH transition policies or preparedness assessments to facilitate the graduation of recipient countries (ie, a shift in their eligibility for DAH).^6 9–16^ These policies are guided by ‘Sustainability Doctrine’, which emphasises capacity building within recipient countries, seeks to prevent aid dependency and backs initiatives that are expected to continue beyond the cessation of donor funding.^3 9 17 18^ In this scenario, maintaining the endline achieved by DAH is synonymous with sustainability,^3 17^ a topic that researchers have comprehensively examined in terms of its outcomes^19–21^ and the factors that facilitate or hinder such ‘sustainability’.^22–25^

Rather than defaulting to the assumption that DAH-supported interventions and programmatic elements should be maintained or scaled up post funding,^26–29^ this study aligns with the views of Cancedda *et al* 3 and Yang *et al* 18 by conceptualising DAH sustainability as the sustained improvement of health outcomes and the mitigation of health inequities following the cessation of DAH support. By focusing on factors contributing to the sustainability of health improvements beyond the DAH period, our study aims to provide insights into how the legacy of DAH can benefit the long-term strengthening of health systems.

To investigate DAH sustainability empirically, we conducted a retrospective case study surrounding the Basic Health Services Project (BHSP; 1998–2007) in China, which received significant support from the World Bank and the UK Department for International Development (DFID, currently the Foreign, Commonwealth and Development Office) (table 1). Despite China’s remarkable economic growth in the 1990s, health systems in the country’s rural areas suffered due to the marketisation of the health sector, a decentralised public health finance system and rural–urban development disparity.^30 31^ While aiming to improve the rural health systems in selected project areas suffering from poverty, the BHSP also served as a policy experiment for China’s health system reforms.^32 33^

**Table 1:** Summary of the Basic Health Services Project

Focus	Rural health system strengthening	
Duration	1998–2007*	
Actual/latest estimate of financing (USD millions)†	World Bank International Development Association credit	98.42
	UK DFID grant	66.6
	Other donors’ grant	3.504
	Chinese subnational counterpart funds	37.51
	Total	206.034
Geographical and population coverage	The project targeted around 43 million people in 97 poverty-stricken counties out of around 660 counties across 10 provinces/municipalities (Qinghai, Henan, Guizhou, Gansu, Anhui, Shanxi, Chongqing, Ningxia, Shaanxi and Sichuan).	
Bottlenecks for the project to tackle	Affordability of health services decreased due to multiple factors, such as the collapsed old cooperative medical scheme, health facility marketisation, excessive drug prescriptions and high service fees and diagnostic test costs. Health service delivery quality, efficiency and effectiveness were poor, reflecting untrained, unsupervised staff and infrastructure investment not aligned with population planning. Rural populations in poverty faced barriers to health services access.	
Project components	Improving planning, management and health infrastructure A1. County health resource plans Health planning training and workshops; (ii) progress reviews and site visits; and (iii) technical support to prepare annual work plans. A2. Upgrading township health facilities Reestablishing and equipping about 1000 township health centres and central township hospitals based on revised service functions and standards. A3. Improving management information system Improving the collection and use of information for planning and monitoring health programmes and the project through (i) improvements to the reporting system; and (ii) surveys. Improving health service quality and effectiveness B1. Improving health service delivery Establishing effective supervision and referral relationships between health system levels; implementing standard case management and infection control protocols and X-ray safety; and piloting essential drug lists to reduce dangerous and unnecessary prescribing. B2. Priority health interventions Improving coverage and utilisation of selected cost-effective interventions, targeting important health problems in project counties. Increasing affordability of health services C1. Cooperative Medical Scheme (CMS) Helping townships to establish risk-sharing schemes. Funds were for startup costs (eg, baseline analysis, scheme design, community mobilisation, management capacity and seed funds). C2. Medical Financial Assistance Scheme (MFA) Establishing an MFA scheme in participating townships, as a means to reimburse service providers in partial payment for health services and inpatient care for the poorest five per cent of households. Project coordination and support	

Source: Project Appraisal Document, Implementation Completion and Results Report, and the China-led final report of the project, with authors’ amendments.

*The project duration was originally 1998–2005 in the project design, but it was extended to 2007. According to the Implementation Completion and Results Report, “the closing date was extended 2 years to allow the implementation momentum to be sustained and project objectives to be met more fully” (see Page 6, World Bank Group. Implementation Completion and Results Report on a credit of SDR 63.0 million for the People’s Republic of China for a Basic Health Services Project [Internet]. Washington, D.C: World Bank Group; 2008. Report No.: ICR512). According to respondents, the reason for the extension was that the committed funds supported by the donors were not used up, especially in the case of less predictable interventions for institutional changes. Little information was about why the project was extended to 2007 not others. Additionally, the World Bank initiated a rural health project from 2008 to 2014, continuing building on the work developed in the BHSP (see Page 7, World Bank Group. Implementation Completion and Results Report (IBRD-75510 TF-92893) on a Loan in the Amount of US$50 Million to the People’s Republic of China for a Rural Health Project [Internet]. Washington, D.C: World Bank Group; 2014. Report No.: ICR2967).

†The funding statistics are inconsistent across project documents. The statistics in this table were extracted from Page 28, the Implementation Completion and Results Report of this project. In the final report published by the Chinese counterpart, the statistics (in USD millions) are: 85 from World Bank International Development Association credit; around 42 (21 GBP) from UK; 4.19 from other donors; and 43.78 from the Chinese subnational counterpart funds. DFID supported the BHSP through a Health 8 Support Project (H8SP), with grants allowing more focus on ‘software’ expenses (eg., those for increasing flexibility of County Health Resource Plans, establishing expert panels, research and compensating transaction costs) as the Chinese government had internal constraints on the use of credit funds. Other donors supported the BHSP through small grants for innovation, experimentation and project implementation strengthening. The costs varied each year as the progresses of different components differed. For example, the Implementation Completion and Results Report revealed that by the end of 2004, with the township health facility program largely completed, the project increased its focus on capacity and institutional building. However, we have not found exact statistics on annual cost.

Through this case, we aim to investigate the integration of DAH-supported interventions into the host country’s health system and its contribution towards strengthening the local health system. Integration, as defined by Atun *et al*,^34 35^ refers to the process by which health interventions, including new ideas, practices, objects or institutional arrangements, are adopted and assimilated into the core functions of a health system.^36^ This process is not limited to the implementation stage but spans the entire lifecycle of a DAH project. Our analysis is particularly interested in how such interventions become an integral part of the local health system, enabling the host country to sustain these interventions independently and in context-specific ways, as opposed to maintaining them as parallel, standalone projects disconnected from the broader health system.

This case study is part of a broader effort to understand the role of DAH in facilitating health system strengthening. Since the 1980s, China has received substantial external health assistance, with international partners significantly contributing to its health system reforms.^37 38^ The BHSP offers critical insights into the effective utilisation of DAH for health system strengthening, a subject that has been comparatively under-researched.^15 39–41^ Despite a wealth of literature and participant reports, there is a dearth of independent research detailing the BHSP beyond the project documentation.^42–44^ This study fills this gap by independently assessing the integration of BHSP interventions into the local health system through the lens of global health.

## Methods

### Study design

We used the case study approach that allows in-depth exploration of complex systems.^45^ From December 2021 to May 2022, we reviewed 64 published papers and policy or project documents, and conducted semistructured interviews with 22 key informants. We used a policy triangle framework built on Walt and Gilson’s model for health policy analysis^46^ and the WHO’s health system building blocks^47^ for data collection and initial coding. From February to March 2023, the final analysis was conducted under the analytical framework developed by Atun *et al* on integrating targeted health interventions into health systems.^36^

### Data collection

This study has adopted a policy triangle framework, which was originally developed for a multicountry research project titled ‘Sustaining Effective Coverage in the Context of Transition from External Assistance—Lessons from Countries’ (The project was sponsored by the Alliance for Health Policy and Systems Research, the WHO, the WHO Department of Health Systems Governance and Financing and UHC 2030).^48^ The framework is consistent with Walt and Gilson’s health policy analysis model,^46^ emphasising four essential components: context, content, actor and process. The WHO’s health system building blocks serve as the foundation for the ‘content’ component.^47^ Specifically, the framework considers the complex interplay of various elements, including the political, economic, institutional and sociocultural context, the content areas of governance, financing, inputs, and service delivery, the array of actors such as the government, development partners, and civil society, as well as the policy-making and implementation processes before, during and after the DAH interventions. Collectively, this approach aims to streamline the collection of comprehensive data pertinent to the DAH initiative. Based on this framework, a data collection tool was customised to fit the unique circumstances of China.

To better understand the BHSP and its context, we searched and selected 64 relevant published papers and project or policy documents (see online supplemental material 1 for the scoping review process and online supplemental material 2 for these documents). The documents include credit agreements, project appraisal documents, progress and completion reports, slides, videos and national policies or strategies on rural health. Most project documents can be downloaded from the World Bank website.^49^ We also obtained completion reports accomplished by subnational governments and slides through personal correspondence. We then identified additional project documents by reviewing the reference lists of related published literature and secured them through online searches. We further identified the relevant policy documents from the already-obtained project documents, related literature and key informants throughout the data collection process.

10.1136/bmjgh-2023-012853.supp1 Supplementary data

Drawing on a preliminary literature and document review, we purposively selected key informants who have been engaged in various stages of the project—namely design, implementation and evaluation—for a minimum of 4 years. These individuals were chosen from diverse backgrounds, including donor agencies, governmental bodies and academic institutions, ensuring a multifaceted perspective. We then encouraged them to nominate additional key informants, thus employing a snowball sampling technique. The outreach efforts were meticulously crafted to achieve an equilibrium between national and subnational stakeholders, predominantly involved in the project implementation, and national and international stakeholders, who primarily contributed to high-level decision-making. Finally, out of the 37 contacted key informants, 22 participated in the interviews—4 were national government stakeholders, 11 were subnational government stakeholders (covering 4 provinces, 1 prefecture, 2 counties and 2 townships), 4 were donor stakeholders, 1 was a stakeholder from an external development partner and 2 were the country’s technical experts involved in the project (see online supplemental material 3 for respondents’ characteristics).

Before each interview, we designed a separate set of guides based on the policy triangle framework and the document and literature review. We then iteratively revised prompts based on previous interviews and background research of the respondents (see online supplemental material 4 for an example of the interview guide). The interviews lasted 1.5–2.5 hours; they were recorded with the respondents’ consent and transcribed promptly in their original languages. Three interviews were conducted in English, and the rest were in Chinese. An additional team member validated the transcripts to ensure their accuracy. We discussed the key findings after each interview.

### Data analysis

To keep the consistency of the abovementioned multicountry research project, we used a framework approach to code the documents and interview transcripts^50 51^ from June 2022 to December 2022, according to the policy triangle framework. Two coders familiarised themselves with the data and conducted a pilot coding for five purposively selected respondents’ transcripts to cover different perspectives. The pilot coding used a deductive approach to test out the major themes in the policy triangle framework and develop a coding scheme for formal coding. In the formal coding, recurrent themes and subthemes were identified by inductively examining the data to develop more specific concepts. We triangulated the results from different data sources to identify patterns and linkages between themes and concepts and explore relationships and explanations. MAXQDA 2022 (Release V.22.3.0) was used for coding.

The research question of this study was framed iteratively throughout the data coding process. We then used the analytical framework for the integration of targeted health interventions into health systems, as developed by Atun *et al* 36 (see table 2). This framework, previously employed in the extant literature on intervention integration within global health,^52^ encompasses various components that may impact integration: the problem, the broad context, the intervention, health system characteristics and the adoption system. These components interact in a dynamic, non-linear way. As this analytical framework is compatible with the policy triangle framework, it proved instrumental in analysing the interactions and interconnections of the data collected surrounding the research question.

**Table 2:** Analytical framework for integration of targeted health interventions into health systems

Component	Explanation
The problem	“The characteristics of the problem will influence the rate at which an intervention designed to address it is integrated into the general health system.”
The broad context	The broad context is “the interplay of the demographic, economic, political, legal, ecological, socio-cultural (including historical legacies), and technological factors in the environment” within which the integration occurs.
The intervention	“Less complex interventions more readily lend themselves to standardization and replication” than complex interventions that “require greater customization to meet the needs of the specific client groups in different contexts”.
Health system characteristics	“Integration can occur at different levels of the health system—local, district, regional or national depending on the prevailing governance arrangements—in relation to critical health system functions”, involving an alignment with the existing system.
The adoption system	The adoption system, within which the intervention is internalised, comprises key actors and institutions “with varied interests, values and power distribution” in the health system and the broad context.

Apart from the data supposedly to be coded based on the policy triangle framework, additional valuable data needed for this analytical framework were further identified through a re-examination of interview transcripts and related documents. These included nuanced details regarding interventions. Finally, framed by this analytical framework, the previously coded themes and subthemes were systematically reorganised, and new themes were identified from the additional valuable data.

## Results

We inductively summarised the collected data on the outcomes of the integration of the BHSP interventions into the country’s health system in table 3 (see online supplemental material 5 for change in coverage for interventions). Based on the analytical framework (table 2), we identified several factors and processes explaining these integration outcomes.

**Table 3:** A summary of BHSP interventions’ integration into China’s health system

Intervention and the integration	Evidence and examples
Various ideas and values were introduced and institutionalised at the national level or in project areas to promote health equity.	According to subnational respondents, the idea of ‘A1. County Health Resource Plans’ took root for some managerial and policy-making officials in project provinces and counties. According to respondents, the project’s approach of government contracting health services was successfully implemented nationwide and accepted to promote government accountability in the health sector since 2009. According to published literature^32^ and respondents, the project advocated the balance of hard versus soft infrastructure* and demand-side versus supply-side investments†, which were routinised in project provinces and counties and included in the discourse of national healthcare system reform in 2009.
Some interventions informed China’s rural health system reforms and were adapted accordingly in national and provincial policies.	According to respondents, ‘B2. Priority health interventions’ tailored to local needs in the project were assumed by provincial public health services and standardised through the National Equalization of Basic Public Health Services initiated in 2009, in addition to other major public health service initiatives. According to project documents,^53 57 58^ published literature,^32 33^ and respondents, the project ‘C1. Cooperative Medical Scheme’ and ‘C2. Medical Financial Assistance Scheme’ were modified to support experimental phases of the New Rural Cooperative Medical Scheme (NCMS) and Rural Medical Assistance (MA). NCMS and MA were launched in 2003 by the national government.
The enhanced health facilities, well-crafted guidelines and protocols, trained human resources and other improvements in health service delivery have boosted the availability and quality of healthcare services, notably in project counties, and have furnished instructive insights for China’s rural health system reforms.	According to respondents, ‘A2. Upgrading township health facilities’ improved the infrastructure and equipment of the township health centres in the project counties, providing valuable lessons for post-project provincial programmes and national primary healthcare facility development. According to respondents, ‘A3. Improving management information system’ and ‘B1. Improving health service delivery’ were routinised in project counties’ health services; B1 provided valuable references for the development of national essential drug lists, guidelines for standard clinical protocols, the development of human resources for health, and public hospital reforms—all as parts of the national healthcare system reform in 2009.

*During the interviews, it was observed that the terms “hard infrastructure” (*yingjian jianshe*) and “soft infrastructure” (*ruanjian jianshe*) were used locally. Specifically, hard infrastructure refers to physical infrastructure such as health facility construction and equipment upgrading, while soft infrastructure refers to personnel training, institutional building, organisational development, policy experimentation and other non-physical aspects of infrastructure development. The collected data revealed that there was a common tendency towards inefficient investment in hard infrastructure over soft infrastructure in China during the 1990s.

†'Demand-side investment’ (*xufang touru*) and ‘supply-side investment’ (*gongfang touru*) were noted as local concepts during the interviews. Demand-side investment adopts a people-centred approach that prioritises improving health services accessibility and expanding medical insurance coverage. Supply-side investment, on the other hand, focuses on enhancing the capacity of health services provision such as health facility upgrading and training for health workforce’s technical skills. The respondents indicated that the lack of emphasis on demand-side investment had been identified as a crucial bottleneck in China’s rural health system during the inception of the BHSP.

BHSP, Basic Health Services Project.

### Favourable context and strategic timing for addressing systemic health issues

**Figure 1:**
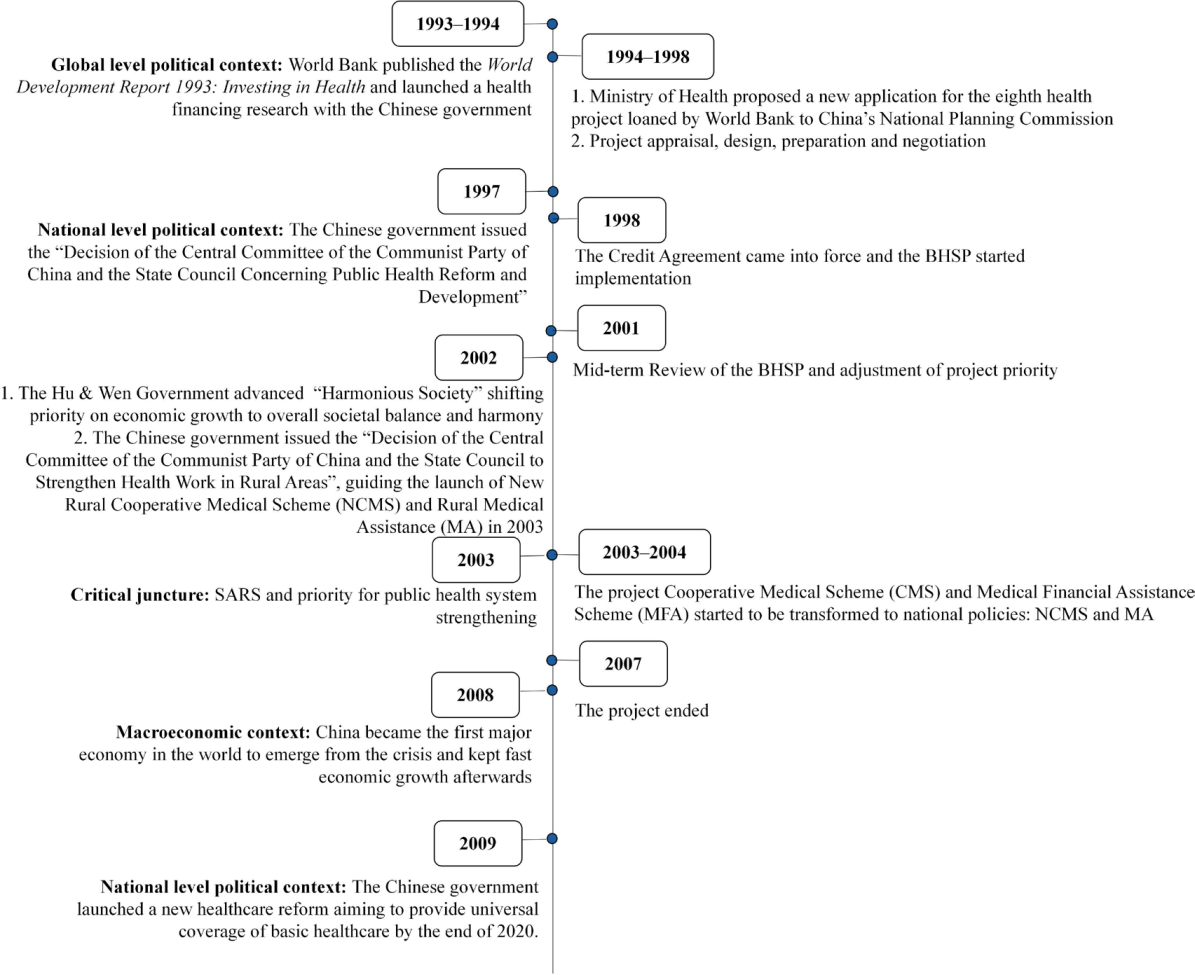
Timeline of China’s critical events of the broad context and project processes. The National Planning Commission (1952–2003) was reorganised into the National Development and Reform Commission in 2003. BHSP, Basic Health Services Project.

The BHSP was set in a favourable context (figure 1), where addressing systemic issues in China’s health sector was crucial due to the country’s transition to a market economy. As the government adopted a pragmatic, experimental approach in its policy formulation, a shift in political priorities towards the health sector and growing economic capacities allowed feasible experiments that could align with the country’s health system needs and be integrated on a larger scale.^33 53^

BHSP is one of the major pilot schemes.^54^ During the project’s inception (1994**–**1998), the State Council issued a decision paper with general principles for public health reform in 1997.^55^ These principles guided the project design,^30^ while the project was perceived as part of the government’s approach to exploring a practical way forward.^32^ Throughout the project years (1998–2007), the country’s central leadership transition resulted in greater political commitment towards improving people’s livelihood and well-being over economic growth. The government launched NCMS and MA in 2003, which had been piloted by the project CMS and MFA. Coincidentally, the SARS outbreak put strengthening the public health system on the political agenda.^56^ Towards the end of the BHSP project, China was already considered an emerging and resilient economy, particularly evident during the 2008 financial crisis. In 2009, the Chinese government launched a healthcare system reform under this promising economic climate. The primary objective of this reform was to enhance basic health services in the country, which resonated with the BHSP.^33^ The reform also integrated some project interventions as routine health system functions. Respondents emphasised the importance of the broad context, with one of them noting that the BHSP integration could have been challenging without this context:

One of the reasons that the BHSP worked is that the timing was right… If someone had tried to do that project three or four years earlier, it would not have worked. And if someone tried to do it in other countries, I’m not sure it worked… And only as the overall economic and political system was able to learn and adapt could this project take place… It’s what we call in donor parlance, particularly in World Bank terms, a “Christmas tree project”—it means that it has all kinds of bells, whistles, and trinkets on everything. Normally those projects are a disaster.—Donor R4

### The intervention characteristics and the corresponding integration outcomes

The BHSP’s interventions were intricate and interrelated. The respondents drew connections between the intervention characteristics and various outcomes of interventions’ integration.

The ideas and values instilled by the project required a supportive context and system for long-term learning and transformative changes in the health system. Respondents reported that principles including ‘promoting government accountability’ (*national government R1 and R2; subnational government R11*), balancing hard and soft infrastructure investments, and prioritising demand-side versus supply-side investments were endorsed by the 1997 public health reform’s policy text.^55^ Project practices and the 2009 healthcare reform integrated these ideas and values across the project areas and the country. However, integrating the concept of ‘county health resource planning’ was limited in the project areas, resulting from this concept’s incompatibility with China’s top-down institutional structure of policy planning that extends beyond the health sector:

For county health resource planning, people felt that it was relatively vague or unclear—it was effective only in project areas but could not be sustained in the long run… Because pushing for reforms is not easy, and it is closely related to our institutional system.—National government R1

CMS and MFA prioritised institutional building, which required beneficiary mobilisation and cross-sector collaboration. Respondents indicated that their integration entailed significant adaptation. Unlike other project priority interventions covering all project areas and populations, CMS and MFA were piloted smaller before the NCMS and MA were launched in 2003. Respondents, project documents^57 58^ and published literature^32^ revealed that before 2003, CMS was initially tested in selected townships of project counties and was once considered a failure. MFA targeted either 20% or 5% of the population in poverty (The project counties were divided into two parts considering different extent of poverty. MFA targeted 5% families in part A counties and 20% in part B counties. UK DFID’s grant supported 8%–10% families in selected pilot counties among part A counties. These criteria were adapted as the project MFA was transformed to national MA since 2004 (see Page 15 in Liu *et al* 58)), but these targets were not reached at the provincial level.^57 58^ It was only after 2003 that CMS and MFA were transformed into NCMS and MA, respectively, with the project counties serving as pioneers of national pilot schemes of NCMS and MA.^33 57 58^ NCMS avoided most of the errors of its predecessor, CMS, by ensuring significant government investment in the institutionalisation of insurance management and achieving more extensive and effective coverage (see online supplemental material 5 for NCMS coverage in the project and non-project counties). Additionally, project elements surrounding MFA were adapted to the national social security system primarily managed by the Ministry of Civil Affairs (MoCA).^33 58^ These adaptations enabled the project to leverage its experiences and resources, making them valuable for national implementation.

With their effects perceived in routine health system activities, interventions like upgrading health facilities (A2), improving health service delivery (B1) and priority health interventions (B2) were viewed as ‘shadows of BHSP’ (*donor R2 and subnational government R11*) in the 2009 healthcare system reform. According to subnational respondents, even between 2007 and 2009, some project provinces continued to support priority health interventions, and most project areas adhered to guidelines and protocols developed by the BHSP in their routine health system activities. Similarly, the tangible outcome of health facilities upgrading by the project was sustained in the project areas following the project’s completion, making the township health centres serve as major service delivery organisations for the rural population.

### Project embeddedness: adapting to health system characteristics

According to project documents and respondents, the BHSP’s service delivery and management systems, including those for finance, procurement and information, shared human resources and infrastructure with China’s existing health system (see online supplemental material 6 for the governance structure), aligning with the government’s strong administrative capacity. This embeddedness largely allowed for the smooth integration of project interventions into routine health system functions with few disruptions.

The project management offices (PMOs) were part of the government. One arrangement was that the existing office performing routine health system functions served as the PMO. Project-designated staff received the same salary as the staff from any other office. This ‘one office, two functions’ approach (*subnational government R2 and R9*) allowed for combining project activities with routine operations and gave the previous PMO managers and staff significant policy influence after the project’s completion. Subnational respondents reported the success of this approach, with most subnational managers we interviewed obtaining high-level positions at health bureaux in the postproject period. This leadership continuity facilitated the further integration of project legacies into the local health systems of the project areas. In two of the four project provinces covered by the interviews, the health bureaux’s Department of Planning and Finance served as the PMO, enabling the BHSP’s experiences to shape further provincial decision-making and practices. Health bureaux doubled as PMOs at the county level, promoting mutual progress between the project and routine operations.

In another arrangement, the PMO operated as a standalone entity. The national PMO, the Ministry of Health Foreign Loan Office, possessed significant expertise in managing projects supported by external development partners, especially since the World Bank began supporting China’s health sector in 1983.^58^ However, respondents reported that few of its managers or staff continued to engage in rural health reforms. Two other provincial PMOs studied also adopted similar standalone arrangements. Some key PMO managers of these two provinces were not involved in policy-making in postproject interventions, probably hindering project interventions’ integration:

Our province’s PMO is standalone—it did not share office space with other departments. We established this PMO and assigned some people to work specifically on the project, so sustaining project interventions was difficult. Currently, all the personnel assigned have been dispersed to various organisations or departments, and there is no one left in the provincial health bureau now.—Subnational government R10

In addition, the absence of the prefectural government was a gap in the alignment of the health system, limiting the potential spread of project experiences at the prefectural level and in non-project counties. According to subnational respondents, project finance was adjusted for effectiveness during implementation, transferring funding directly from project provinces to counties and thus bypassing the prefectures. In addition, as project activities diverted resources for the routine functions of prefectural governments, their involvement gradually faded. Furthermore, although the spread of project experience to non-project counties was part of project activities, it was merely marginal.^57 59^ Thus, the BHSP had rather indirect policy influence related to the 2009 national healthcare system reform at prefectural levels and in non-project counties.

Another important aspect of project embeddedness was the cofinancing arrangement between the government and donors, which positively and negatively impacted project implementation and intervention integration. On the negative side, the arrangement financially burdened subnational governments. They were required to provide all the counterpart funds.^32 60^ However, these subnational governments could only meet 84.9% of the initial estimate due to their impoverished status.^57^ As the resource-constrained subnational governments preferred investing in health facility upgrading, investment in ‘soft infrastructure’ (*donor R1 and R2; national government R2; subnational government R2, R3, R8 and R9*)—capacity and institutional building—was compromised,^57 61^ together with the compromised integration of these interventions. On the positive side, project managers dedicated considerable effort to advocacy for project interventions with government leaders to gain political support and sufficient counterpart funds. This advocacy helped strengthen local ownership and commitment conducive to integrating project interventions. Subnational respondents and a project report^57^ attested to this positive impact.

Through providing counterpart funds, we reported [the project progress] to the [government] leaders, and they spontaneously knew that someone was carrying out the project interventions and thus gave us money. They had to pay attention to these interventions.—Subnational government R4

### Adoption system: role of project leading groups and technical support

As part of the adoption system, the project leading groups (see online supplemental material 6) at the national, provincial and county levels were critical in strengthening political commitment and cross-sectoral collaboration. According to respondents and project documents,^30 57^ the leading groups mobilised related ministries and departments (including those for health, finance, civil affairs, etc), raising the project’s visibility among the sectors involved. At the subnational levels, the leading groups were chaired by executive leaders of the subnational governments,^57 58^ who were responsible for supervising the project implementation at the subnational level. Especially in the semiannual project supervisions, these subnational government leaders worked with project managers and experts at the subnational, national and international levels for project problem-solving.^59^ Respondents reported that the project leading groups and the according supervisions elevated the subnational government’s attention to the health sector and the project interventions’ sustainability:

The technical support system (see online supplemental material 6) played a vital role in these supervisions as well as the adaption of international norms and practices through evidence-based research and consultancy.^32 57^ ‘Mutual learning’ characterised the relationship between international and local experts.^32 59^ Through the interplay of international and local knowledge and frequent fieldwork in the project, international and local experts jointly helped to adjust the project activities to suit the ever-changing local needs. They also developed innovative approaches that aligned with China’s context.^32 62^ Under this technical support system, donors insisted on a need-based approach for systemic improvement in the negotiation, ensuring local adaptation and recipient leadership in the project.

Respondents highlighted the example of MFA adaptation. Given the subnational governments’ preference for facility upgrading over institutional building, MFA preparedness became a precondition for receiving credits for facility upgrading, ensuring MFA implementation. A national expert hammered out this ‘setting precondition’ approach, while the international experts primarily introduced the concept of medical assistance.^44^ Moreover, when the national MA was in its initial stage around 2003, there was a major change in MoCA personnel. To introduce project lessons and models to the new MoCA personnel, local project experts and DFID experts jointly worked to design and evaluate MoCA’s MA pilots in BHSP project counties.^32^ This technical support system penetrated at every level and highlighted the empowerment of local experts, ensuring contextualised adaptation and facilitating the integration of project interventions into the local health system.

It Is important to involve local experts to achieve sustainable development. After our project ended and international experts left, knowledge, theories, and concepts were maintained in the country. These were applied into practices and promoted further by local experts.—Donor R1

## Discussion

The case study of BHSP reveals several factors of integration of DAH-supported interventions into the local health system, indicating pathways for DAH sustainability.

First, the project was aligned with the country’s health system needs and its broader development trajectory under a supportive broader context, including a growing political commitment to public health, a robust economic environment and a policy culture favouring experimentation and learning. This finding corroborates that of prior research,^24 63^ while also delivering a more nuanced and comprehensive narrative.

Second, the intervention characteristics might link to different integration outcomes. These outcomes can vary by the levels of integration (national or subnational), geographical coverage (project areas or both project and non-project areas) and approach to integration (policy or routinisation). Long-term learning and transformative changes are needed for value-driven interventions, but resistance can occur if the changes are subversive. Institutional building interventions require significant adaptation, as seen with CMS and MFA, while those with tangible outcomes, like health facility upgrades, are more easily accepted. This reflects criticisms of global norm diffusion models,^64 65^ emphasising that DAH integration can take different forms to achieve sustainable health outcomes, including resistance, acceptance, modification, innovation and expansion.

Third, the results of this study also support the argument that project embeddedness into the existing system is conducive to DAH transition^6 9^ and sustainability,^23 24 28 66–68^ using the evidence of the ‘one office, two functions’ approach in PMO arrangement and cofinancing in resource-constrained settings.

Lastly, the results on the adoption system are consistent with previous literature on DAH sustainability that attaches importance to ‘bringing global and local expertise and knowledge to bear’.^69^ Project visibility and efforts to prioritise the health sector among government leaders are vital to strengthening local ownership for integrating adapted health interventions. While project visibility might link with external influence and donor–recipient power asymmetry,^70 71^ this asymmetry can be mitigated through project arrangements (eg, a technical support system in this case) that favour mutual learning and knowledge translation between international and local partners.^48 72–76^

This study has turned to project design and implementation to investigate the integration of DAH-supported interventions into the host country’s local health system. Regarding transition preparedness,^16 77–79^ respondents commented that there was no predefined transition period. The project concluded in 2007, given that the funds were used up and the interventions were completed as designed. On termination, the project prioritised dissemination activities to document and spread its experience, and some interventions were refined and designed as components of a subsequent World Bank project.^80^ However, there were inadequate mechanisms to ensure local ownership of the interventions. Additionally, given that Chinese counterparts were primarily responsible for implementing project implementation, donor–recipient power dynamics^15 81 82^ were more pronounced in decision-making during the initial project design^44^ and the country’s broad policy formulation,^33^ extending beyond the specifics of intervention integration. The principal implication of this study is that DAH sustainability can be promoted within the scope of the project. Accordingly, this study highlights several practical implications for DAH transition and sustainability, which necessitate continued engagement from the stakeholders of the host country and development partners alike during the project’s lifespan.

1. Thoroughly assess the conducive and obstructive contextual factors and the potential for policy convergence to maximise the likelihood of successful integration and sustainability, although these factors are typically beyond the control of the DAH project.^24^ Development partners can foster long-term engagement with locally driven country sector studies, as exemplified in the World Bank health financing research in figure 1.
2. Carefully design the intervention characteristics and envision the integration outcomes, considering the intervention’s compatibility with the local institutional system, local health system needs, the political commitment of key stakeholders and the intervention scale feasible for development partners.^83^ Integrate the interventions in the most beneficial form for and compatible with the local health system.
3. When determining the appropriate level of project embeddedness into the existing system, consider the project’s nature and the local health system’s characteristics. It may be advantageous to leverage the existing system’s resources and ensure that project governance aligns with all levels of the country’s health system.
4. By setting cross-sectoral leading groups and boosting mutual learning of international and local technical experts, projects can cultivate the project visibility among key decision-makers and promote dynamic local adaptation.

The study has several limitations that may affect its transferability. As a single-country case study focusing on a project within a specific timeframe, it could only provide a contextualised perspective on the rural health system in China during that period. Moreover, as the analytical framework limits the study scope to project interventions, primarily design and implementation, further research is needed to understand the political dimensions of DAH funding and policy interactions between the host country and development partners.

Methodologically, this study is retrospective. Unlike a normal programme evaluation, the study had to explore the availability of pretransition data largely dependent on the project’s data collection and preservation and then link the project data with the postproject data. However, the country’s health system has undergone epistemic changes after the project’s completion. Still, we have presented as much data as we could collect in this article and online supplemental material 5. Furthermore, the interviews were restricted by time and resources and did not reach stakeholders outside the health sector. Community or beneficiary stakeholders were also not involved in the interviews, as our data provided limited information on community and beneficiary participation concerning the integration and sustainability of project interventions. In addition, there may be recall bias among the respondents. Despite these limitations, the study involved a broad range of stakeholders and achieved data saturation, considering the perspectives of stakeholders we could contact.

## Conclusion

Using the case of BHSP supported by the World Bank and the UK in China (1998–2007), this study claims that the DAH-supported interventions can achieve sustainability by being integrated into the local health system. This integration can take various forms to improve health outcomes, including being accepted and internalised, modified, as well as innovated and expanded, if not being resisted. Stakeholders of the host country and development partners can promote DAH sustainability within the scope of the project by assessing the conducive and obstructive contextual factors and the potential for policy convergence, carefully designing the intervention characteristics while integrating the intervention in the form that is the most beneficial for and compatible with the local health system, determining the level of project embeddedness within the existing system based on the local health system’s characteristics, cultivating project visibility among key decision-makers and promoting dynamic local adaptation through international–local expert collaboration. Future research can focus on the integration of DAH-supported interventions in other settings and the role of political dynamics between the host country and development partners in achieving DAH sustainability.

## Data Availability

Data are available upon reasonable request.
