# Supplementary material for: Integrating interventions supported by development assistance for health into local health system: evidence from a China–World Bank–UK rural health system strengthening project (1998–2007)
Source: BMJ Glob Health. 2024 May 24;9(5):e012853. doi: 10.1136/bmjgh-2023-012853 (PMC11129031; doi:10.1136/bmjgh-2023-012853)
Supplement: Supplementary data [file bmjgh-2023-012853supp001.pdf]

Supplemental material 1. Scoping review process

We conducted a scoping review of related published academic literature as well as policy and project report/documents that analyzed the design, implementation, and influence of medical financial assistance of the Basic Health Services Project (BHSP) in China. The objectives of this review were (1) to identify existing sources documenting the BHSP, (2) extract data for the research project 'Sustaining effective coverage in the context of transition from external assistance – Lessons from China', and (3) support analysis for sub-themes of this broad research project, such as 'sustainability of development assistance for health' in this study. As we aimed to conduct case study on the BHSP and found few studies summarizing literature and documents on these projects, we believed a scoping review necessary for investigating the two projects comprehensively.

We combined terms and phrases related to World Bank, DFID, and the BHSP on Embase, MEDLINE, and China National Knowledge Integrated Database (see search strategy below). We also conducted additional searches on Google, Google Scholar, World Bank Open Knowledge Repository and eLibrary, the Overseas Development Institute, and the official websites of the World Bank and the UK government. We also identified additional studies or documents by reviewing the reference lists of related published literature and secured them through online searches. The search limited the publication date from 1995 to 2020. Some studies or documents were obtained through personal correspondence, which might be published after 2020. Throughout the data collection process, the relevant policy documents were identified as the project documents, related literature, and key informants indicated some of them.

We included all studies if they analyzed each specific project examined or are relevant to the general topics that could be helpful to our understanding of the transition context (e.g., analyzing the role of World Bank and DFID in China's health system reform); we excluded studies if they did not mention the specific projects or have a very limited analysis of them that do not contribute to our understanding of the project design, implementation and transition. We conducted two rounds of screening, first title and abstract and then full-text screening, and all screening was conducted by one team member whereas uncertain ones were discussed with another team member. A total of 4452 citations were screened.

Finally, we included 64 studies and documents for this study. Data charting followed the policy triangle framework while data synthesis has been scored in MAXQDA, triangulated with data from our interviews. As this study's focus is not the scoping review, and a long report of the review process will distract the readers' attention, we highlight a list of major literature and documents in supplemental appendix II. In the same vein, as this scoping review aimed not to report the sources per se, but to extract secondary data for the qualitative research mentioned above, 'results' and 'discussion' in the PRISMA for scoping reviews are basically not applicable here.

Search date: February 4, 2022

Embase and MEDLINE through OVID

#	Searches	Results
1	("World Bank" or "WB" or "IDA" or "International Development Assistance" or "DFID" or "Department for International Development" or "China").ab,kw,ti.	554507
2	("Basic Health Services Project" or "Health VIII" or "H8SP" or "Health Eight Support Project" or "H8/SP" or "Qinba Health program").af.	42
3	1 and 2	5
4	remove duplicates from 3	3

China National Knowledge Integrated Database (“中国知网”)

Result: 1315

Searches: ((((旧版主题= '世行'+'世界银行'+'世界银行贷款') AND (旧版主题= '卫八'+'卫生VIII'+'卫生VIII项目'+'基本卫生服务项目'+'秦巴卫生项目'+'卫生VIII支持性项目')) OR (关键词='卫生VIII项目')) NOT (文献来源%'年鉴' or 中文刊名%'年鉴')) AND (发表时间 Between('1995-01-01','2020-12-31'))

Language: Chinese

Google and Google Scholar

Search strategy		Results	
		Google	Google Scholar
Searches (English)	(“World Bank” OR “WB” OR “IDA” OR “International Development Assistance” OR DFID OR “Department for International Development” OR China) AND (“Basic Health Services Project” OR “Health VIII” OR H8SP OR “Health Eight Support Project” OR H8/SP OR “Qinba Health program”)	83	54
Searches (Chinese)	(世行 OR 世界银行 OR 世界银行贷款) AND (卫八 OR 卫生VIII OR 基本卫生服务项目 OR 秦巴卫生项目)	123	836

Filtered year	1995-2020	
De-customized	Yes	

World Bank websites

Website	Searches	Results
World Bank Open Knowledge Repository	China AND (“Basic Health Services Project” OR “Health VIII” OR H8SP OR “Health Eight Support Project” OR H8/SP OR “Qinba Health program”)	1111
World Bank eLibrary	China basic health services	629

Overseas Development Institute

Searches	Results
basic health services project China	7
Health VIII China	1
Health Eight Support Project China	113
H8SP, H8/SP, Qinba health program	0

Official websites:

1. World Bank Projects & Operations

   https://projects.worldbank.org/en/projects-operations/project-detail/P003566

2. UK government

   https://www.gov.uk/search/all?keywords=basic+health+services+project+China&level_one_taxon=37d0fa26-abed-4c74-8835-b3b51ae1c8b2&level_two_taxon=9fb30a53-70fb-4f1c-878b-0064b202d1ba&order=relevance&page=1

Supplemental material 2. Major project and policy documents and multimedia

Project documents

1. World Bank Group. Project Appraisal Document on a proposed credit of SDR 63.0 million for the People's Republic of China for a Basic Health Services Project [Internet]. Washington, D.C: World Bank Group; 1998 Apr. (World Development Sources). Report No.: 17403. Available from: http://documents.worldbank.org/curated/en/406281468769778128/China-Basic-Health-Services-Project

2. World Bank Group. Implementation Completion and Results Report on a credit of SDR 63.0 million for the People's Republic of China for a Basic Health Services Project [Internet]. Washington, D.C: World Bank Group; 2008. Report No.: ICR512. Available from: http://documents.worldbank.org/curated/en/293281468028733713/China-Basic-Health-Services-Project

3. Liu Y, Liu G, Liu M, Xu L, editors. Jiaqiang Zhongguo Nongcun Pinkun Diqu Jiben weisheng Fuwu Xiangmu Wangong Zongjie Baogao [Final report on China Basic Health Services Project]. China Financial & Economic Publishing House; 2007.

   刘运国、刘谷琮主编：《加强中国农村贫困地区基本卫生服务项目完工总结报告》，北京：中国财政经济出版社，2007 年 6 月。

4. Legal Dept of World Bank. Conformed Copy - C3075 - Basic Health Services Project - Development Credit Agreement [Internet]. Washington, D.C: World Bank Group; 1998 Jun. Available from: http://documents.worldbank.org/curated/en/739291468010836058/Conformed-Copy-C3075-Basic-Health-Services-Project-Development-Credit-Agreement

Policy documents

1. Central Committee of the Communist Party of China and State Council of China. Zhonggong Zhongyang Guowuyuan Guanyu Shenhua Yiyao Weisheng Tizhi Gaige de Yijian [Opinions of the CPC Central Committee and the State Council on Deepening the Health Care System Reform] [Internet]. Mar 17, 2009. Available from: http://www.china.org.cn/government/scio-press-conferences/2009-04/09/content_17575378.htm

《中共中央、国务院关于深化医药卫生体制改革的意见》（2009 年 3 月 17 日）

2. Central Committee of the Communist Party of China, State Council of China. Decision of the Central Committee of the Communist Party of China and the State

Council Concerning Public Health Reform and Development [Internet]. Jan 15, 1997. Available from: http://www.asianlii.org/cn/legis/cen/laws/dotccotcpocatsccphrad1148

《中共中央、国务院关于卫生改革与发展的决定》（1997 年 1 月 15 日）

3. Central Committee of the Communist Party of China, State Council of China. Zhonggong Zhongyang Guowuyuan Guanyu Jin Yibu Jiaqiang Nongcun Weisheng Gongzuo de Jueding [Decision of the Central Committee of the Communist Party of China and the State Council to Strengthen Health Work in Rural Areas] [Internet]. Oct 19, 2002. Available from: http://www.gov.cn/gongbao/content/2002/content_61818.htm

   《中共中央、国务院关于进一步加强农村卫生工作的决定》（2002 年 10 月 19 日）

Multimedia

1. China Central Television. CCTV-Yangguang Zhaojin Shangang [CCTV-The Sun Shone into the Hills] [Internet]. 2007 [cited 2022 Feb 5]. Available from: http://discovery.cctv.com/special/C18692/01/index.shtml

   中国中央电视台：《阳光照进山岗》，央视一套《见证·亲历》，2007 年 6 月 16 日至 21 日。

Supplemental material 3. Respondents' characteristics

**Respondent Number**	**Project design and planning**	**Project management and implementation**	**Not directly involved in the project***
National government R1, R4	√	√	
National government R2		√	
National government R3	√		
Experts R1-2	√	√	
Donor R1	√	√	
Donor R2-4	√		
Partner R1			√
Subnational government R1-6	√	√	
Subnational government R7-9		√	
Subnational government R10-12	√	√	

Note:

** Not directly involved in the project: the respondents involved in the project evaluation, policy advocacy or post-transition programs for sustaining the project priority interventions.*

Supplemental material 4. An example of the interview guide

1. What is your position and role in the health sector? What was your involvement with the BHSP project?

2. How was the planning of the BHSP coordinated with the country health system and national health strategy?

3. Sustainability plan: Was a sustainability plan developed?

If yes:

- By whom was it designed?

- How did it define sustainability?

- What did it plan sustainability of interventions supported by the project after the project ends?

4. Transition period: did the project have a “transition” period?

If yes:

- What criteria were used to define the transition period?

- How were those negotiated? - By whom?

- How was the timing decided and by whom?

- How was transition designed?

- Who was involved in the transition planning process? Who were the stakeholders engaged in the decision to transition donor funding? Was there involvement across sectors?

- Were those expected to take over the project activities, involved in the transition design and implementation?

- Who were actors engaged in either the implementation of the transition policy/supported programs and what were their roles?

- What roles did government, civil society, and development partners play in this process? How did this influence the evolution of the project’s content?

- How did the transition process influence what was finally transitioned in terms of each health system function and how and why did institutions take over or not take over the functions initially envisaged?

- How did the transition process prepare key actors for the adaptation of functions previously supported by external funding? Did this include a concerted effort to build the capacity of national institutions?

5. Governance

- What was the level of commitment from the political level for the project? Did this vary between the pre and post transition periods?

- What was the budgeting process for the project?

- Was there a donor coordination arrangement that worked with the MoH? What role did this play, and how did this vary pre and post transition?

- What's the role of project management office in transition?

- What were the accountability mechanisms within the project?

6. Financing

- What was the source of funding for this project at the starting point? Was there more than one source? If so in what proportion were different sources providing funding? How did the funding source/s of different components change post transition?

- To what extent have financial responsibilities been transferred from World Bank/DFID to China?

- How was domestic financing for the intervention scaled up? What is the balance between domestic government funds and out-of-pocket payments?

- To what extent has the entity implementing the project (esp. the subnational one) post transition secured adequate funding to sustain interventions?

- To what extent were the budgetary and financial systems of the project pre transition, aligned with those of China? How did this evolve over time?

7. Inputs

- To what extent were there technical, managerial, and financial capacities within China to effectively deliver key health program services? How does this compare between the pre-transition and post transition periods?

- Were consumables and pharmaceuticals for the project procured, stored and distributed through dedicated supply chains or did the project use general domestic systems? How was this different between the pre and post transition periods?

- How were data generated, managed and used by the project? Were information systems for the project separate or were they integrated with broader health information systems? How was this different in the pre and post transition periods?

- To what extent were information systems actively used and by whom to make decisions such as those around human resource management, supply of drugs and

other consumables and delivery of services for example. How did this vary across the pre and post transition periods?

- To what extent did the projects M&E systems align with China's M&E systems, including indicators and reporting periods? How did this change between the pre transition and post transition periods?

- What kind of support for human resources for health was present in the project pre-transition? How did this change post transition?

- Did the project have dedicated human resources? How did the project compensate HR? Did this vary between pre and post transition periods?

- To what extent were reporting structures for human resources in the project pre-transition aligned with those of China? How did this evolve during the process of transition and after transition?

9. Transition results and factors

- What do you think of transition results of the BHSP? How have key outcome indicators and health outcome indicators relating to the donor-supported interventions changed? How has the effective coverage of project-supported interventions changed after transition? What is the impact of project transition on the crude coverage of services previously covered by external funding?

- How did intervention, donor and recipient characteristics influence transition?

- How did political, institutional, economic, socio-cultural context influence transition?

10. Others

- What's your overall observation on transition of external assistance in China?

- Who would you recommend as the key informant(s)?

- Any documents or data sources recommended?

Supplemental material 5. Change in coverage for interventions

Project component	***Changes in spending***	***Coverage indicators and outcome at project completion (2007) in project counties***	***Post-project coverage indicator and outcome (national level*)***
County Health Resource Plans (CHRP)	No earmarked funding post-project	# (%) counties with approved CHRP and annual review: 71 (100%)	No data**
Upgrading of Township Health Facilities	It depends on the revenues of Township Health Centers (THC)/Central Township Hospital (CTH) and their application for funding from provincial or national programs	• # (%) upgraded facilities according to CHRP: 95 (100%)<br>• Bed occupancy rate of THCs: 51.1%; of CTHs: 63.0%<br>• Ratio of outpatient visits relative to professional staff in THCs: 3.2; in CTHs: 2.9	Bed occupancy rate of THCs: 55.8% (2008), 64.3% (2013), 53.6% (2020)
Improving management information system	Increasing nationally together with healthcare reform in 2010, which emphasized health information technologies	(%) of counties/towns with an information system working to stipulated standard reports: 100%/85%	No data, but with data of an indirect indicator, health management information system (HMIS) digitalization rate of rural and urban areas: 50.2% (2011), 83.8% (2017)
Improving health service delivery	N/A as the interventions in this component were institutional and hard to measure financing post-project	Selected indicator***:<br># (%) THC and village clinics using essential drugs: 81%/84%	No data. But the essential drugs list has been used nationwide since the 2009 healthcare reform.
Priority health interventions	15 RMB (~$2) in 2008 and 74 RMB (~$11) in 2020 on average per person per year nationally	Selected indicator:<br>Coverage of HepB: 67%	Newborn HepB vaccination coverage (≥3 doses): 99.7% (2012)

Cooperative Medical Scheme	Government financing and its proportion in total NCMS financing on average per person per year nationally: 40 RMB (~$5.8, 80%) in 2008; 380 (~$54, 76%) in 2015	• People enrolled in CMS: 10,236,498<br>• % Townships where CMS schemes met agreed conditions for project support: 90% started; approximate 35% kept compliant till the end	The national rural cooperative medical scheme participation rate: 85.96% (2007), 98.9% (2015)
Medical Financial Assistance Scheme	Medical Assistance financing per person per year nationally: 70.18 RMB (~$10) in 2006; 249 RMB (~$35) in 2014	Selected indicator:<br>% The eligible poor population receiving MFA: 90%	The major disease insurance scheme covered 100% population in poverty in the country (2017)

Source: Authors based on (7,15)*, National Yearbooks and interviews.*

*Note: * Given the complex nature of the BHSP, most project-specific indicators were not used or measured by the national health management information system (HMIS) post-project. As the project counties only accounted for around 10% of counties in each province/municipality, and outcomes like the percentage of hospital deliveries and infant mortality rate might be attributed to factors outside of the project, outcome data will be meaningful only if they are county-level. Unfortunately, we failed to access the county-level outcome data covering project and post-project periods spanning over five years.*

*** Coverages of components like CHRP, improving management information systems and improving health service delivery were hard to measure as they were either institutional or without indicators in the HMIS.*

**** Selected indicator: the corresponding component had a list of project-specific indicators, and in this table, we selected indicators with post-project evidence.*

Among the country's common healthcare indicators related to BHSP interventions, the authors only managed to obtain longitudinal data on New Rural Cooperative Medical Scheme (NCMS) participation rates in the project and non-project counties (Supplementary Figure 1), which became available after 2004. The values for non-project counties present considerably greater variation. Around 2011, there was a notable turning point where most project counties showed a participation rate exceeding 90%. Therefore, there are similar trends between project and non-project

counties, indicating that the influence of the project may be less discernible.

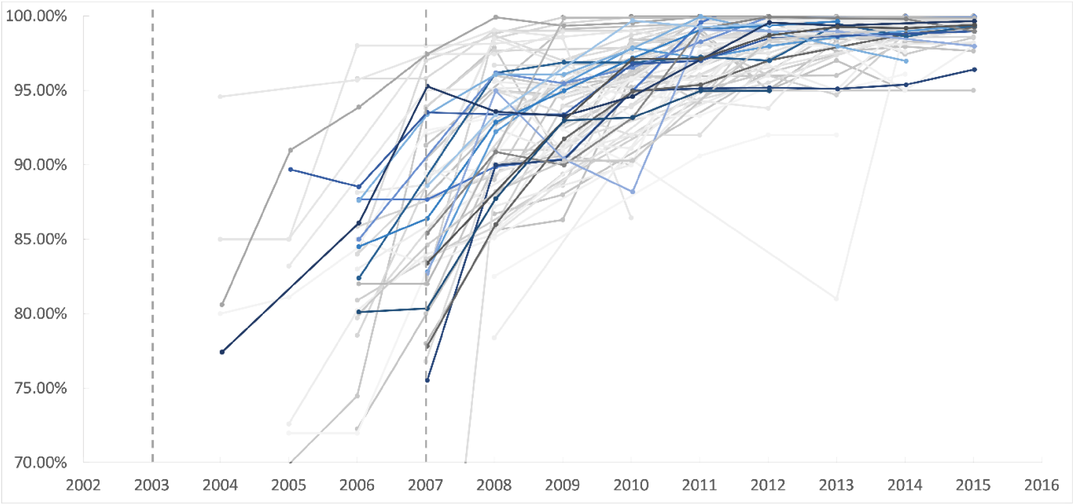

Supplemental figure 1 Participation rate in the rural cooperative medical scheme, data from 18 project counties (blue lines) and 36 non-project counties (grey lines), 2004-2015

Source: Author based on Provincial, Municipal or County Yearbooks

Note: Blue lines represent 18 project counties with available data. The same shade of colors means the counties in the same provinces/municipality. Grey lines represent 36 non-project counties selected randomly in the list of national poverty counties.

<u>Quantitative data mapping</u>

The authors conducted quantitative data mapping and analysis for the BHSP. The study's interest was the county-level data, as the project targeted poverty counties to improve rural health. The authors tried to extract NCMS coverage; the number of health facilities, beds in health facilities, and medical staff; neonatal and maternal mortality; and the government health budget and total budget. However, only NCMS coverage had longitudinal data covering over five years and spanning across pre- and post-project periods. The authors then extracted available NCMS data from 18 project counties (from 6 provinces, 30% of all project counties). The authors also screened and extracted data for non-project counties in the same provinces as the comparison group. 142 non-project counties have data for NCMS coverage over five years; however, as the authors could not extract all of them due to limited time and resources, they randomly selected 36 counties (1:2 matching with project counties in each province) for extraction. The steps of NCMS data extraction are shown below.

1. CNKI yearbook advance search: https://kns.cnki.net/kns8/AdvSearch?dbcode=CYFD

2. Key words (in Chinese): FT=xx*"Cooperative Medical Scheme" AND TI=xx, explode: synonyms expansion, year: 2001-2016, item type: exclude "long-term plan", "people", and "laws and regulations". xx stands for the county's name.
3. Sorting by year to see whether the target county has five years of yearbooks. If not, move to the next county.
   Note: Ensure to double-check the search results in order to avoid inaccuracy due to the ambiguous nature of county names.
4. Extract data from the same series of yearbooks to avoid trivial issues (e.g. rounding errors).

Supplemental material 6. The management structure of the Basic Health Services Project

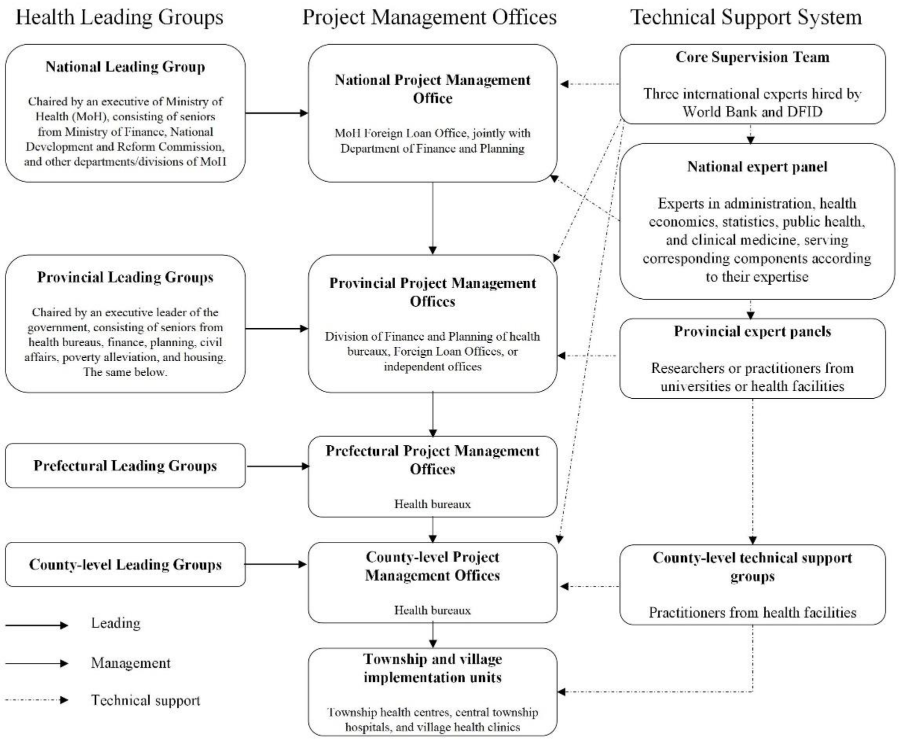

Source: Implementation Completion and Results Report (38) *and a project report* (39)*, with authors' amendments*
